# Bethlem and Insanity

**Published:** 1910-08-13

**Authors:** E. G. O'Donoghue

**Affiliations:** Chaplain to Bethlehem Hospital.


### SPECIAL ARTICLES.

### BETHLEM AND INSANITY.

### THE EVOLUTION OF THE MODERN MADHOUSE.

By the Rev. E. G. O'DONOGHUE, Chaplain to Bethlehem Hospital.

The earliest allusion to the treatment of the insane at Bethlehem is to be found in an inventory of property which had been placed in the hands of the janitor of the hospital some years before 1400. This man, Peter Taverner, was, fortunately for the historian, a thief and a rogue, a drunkard and a gambler. Among the articles which he stole, or allowed tramps to steal, were "six chains of iron with the locks and keys belonging to them, five other chains of iron, four pair of iron manacles and two pair of stocks." There is no whip in the catalogue, but the swish of the lash was common enough in contemporary psychiatrical practice. In one of the windows in Canterbury Cathedral there are paintings which give us a glimpse of mediæval Bethlehem. In one panel the patient is made to kneel at the tomb of St. Thomas à Becket, while two men, obviously keepers, are preparing to beat him. In the alternate panel he is quietly kneeling before the shrine, while one of the keepers lifts up his hands in astonishment. The cords and whips lie on the ground, no longer needed. It is possible that this was a case of acute mania, in which whipping was a common prescription. Perhaps something like convalescence preceded the visit to the wonder-working grave.

#### Bedlam in Literature.

The whip cracks often enough in Bedlam when Jacobean literature begins to haunt it for characters and types. Dekker, to illustrate the survival of the whip, inserts whole scenes from Bedlam in his dramas, though it is his pleasant humour to put in the programme, "Scene: Milan." In one of his plays ("The Honest Whore") the threat of the whip saves the visitors from an attack by the cowed and cowering maniac, who had identified them with his persecutors. In "The Changeling" (Middleton) one of the patients, as he hears the "chimes of Bedlam go," cries out: "See the wire comes," from which it is reasonable to infer that the whip was sometimes a sort of cat-o'-nine tails with metal tags. In Bethlehem Hospital then the whip would also have been employed in its early centuries for the benefit of keepers and visitors, to instil just that sense of fear which preserves the tamer in the den of lions. This, by the by was the philosophy of the Rev. Francis Willis, who suffered, or encouraged, the keeper to knock down his sacred Majesty George III. "as flat as a flounder" when he threatened to be troublesome.

But whips and chains and stocks were only part of the treatment of insanity in the Middle Ages. In cases of religious insanity, in obsession, and in melancholia, where there was loss of memory, confusion, or loss of identity, the weapons of the spiritual armoury would naturally be employed by ecclesiastics. The service of exorcism was as usual as in Babylon or Egypt, or prayers were offered up by the "possessed" and his family to a patron saint of the mad—St. Vitus, St. Fillan, or St. Dymphna (of Gheel); or a pilgrimage was advised to a holy well or popular shrine. Let me illustrate the religious treatment of insanity by a story extracted from the "Foundation of St. Bartholomew's," translated by Dr. Norman Moore. We may admit that people were somehow healed, while we may feel uncertain as to the ingredients of the healing draught—the atmosphere of faith and responsive emotion—the *vis medicatrix* of Nature and time—and the purging and the bleeding.

The date of the following narrative is more than a century earlier than Bethlehem's birthday (1247), but one example will suffice for all the class:—

"A certain young and comely person, and of good breeding, Robert by name, proposed to journey from Northampton to London, and it happened to him that, as he sought to take rest in a thick wood, he was entangled in the snare of the Enemy. For in his sleep he was ravished out of his wits and reason, his old Enemy appearing to him in the form of a very beauteous woman. For a good while she sat at his head, and caressed and petted him. Then she put a little bird in his mouth, and vanished out of his sight. The man, on awaking, was full of fear at so strange a vision, and the same hour he lost his wits and reason, as well as all power over himself, and he knew not what to do or what not to do. In his madness he wandered now this way and now that; unconscious of what he did, he hurried whither the impulses of his madness led him. In the end he was detained in London, and was brought to the church of St. Bartholomew, and there in a short space of time he recovered his reason. And he tarried there a little time, blessing God, who had vouchsafed to commit to His Apostle his excellent power to heal the sick, to cleanse the leper, and to cast out devils."

#### Exorcism.

In this instance and in all such cases the service of exorcism would be an inevitable and familiar preliminary. There are those who even to-day wish to see it revived in asylums. It was sanctioned by the Church of England as late as the reign of James I.; one of the canons of 1603 forbids the casting out of spirits without special licence, in consequence of many amateur exorcists. Shakespeare, in his "Comedy of Errors," Act IV., scene 4, has a reminiscence of the formula of exorcism, which was no doubt pronounced by the monks of Bethlehem as long as there were any: "I charge thee, Satan, housed within this man, to yield possession to my holy prayers." Possibly also—for this was an integral part of the performance elsewhere—a patient of Bethlehem had also to sit on an operating chair, duly hallowed, and had to "take a pint of sack and salad oil, while a pan of burning brimstone was held under his nose." This method of treatment, a version of which Bunyan witnessed in 1682, would produce vomiting anyhow, and, after all, it was less dangerous than being left to sink or swim in a holy well; this was a custom in Cornwall. We must not imagine, however, that in the Middle Ages the dose of medicine had no place of honour. Here is a prescription for "wodness" (madness):—"Take gentian and the seed of rue, boil them well together, and give to drink. Then slay a black cock, and bind it all about the head, and let the patient lie so all day and all night, and on the third day let him bleed on the forehead, and he shall fare well." And with the monastic knowledge of herbs the sleeping-draught would be in nightly use:—

> To provoke repose in him
> Are many simples operative, whose power
> Will close the eyes of anguish.
>
> King Lear, iv. 4.

The wills of the fifteenth century—for example, those of Gower, the poet, and of Carpenter, the town clerk of London—record sums of money bequeathed to be spent on clothing and blankets for the "poor mad folk," or "prisoners" in the "House or Hospital of our Lady of Bedelem." But it is not until 1519 that we get any further evidence in contemporary documents as to the medical treatment of the insane. There is in the British Museum a Certificate of Admission into the Confraternity of St. Mary of Bethlehem. It is in black letter, and still displays a fading woodcut of the Star and the Magi. It recites a list of indulgences granted by six of the Popes to "all who render help towards the support of the mentally afflicted, the insane, the frenzied, who are lodged and cared for in the hospital with great diligence and are treated by the physicians with unceasing solicitude."

The spiritual sway of these pontiffs ranges between 1219 and 1492, and the indulgences are bestowed upon the mother-house of Bethlehem in Palestine, and upon all the daughter-houses in London and elsewhere. It is possible, therefore, that the monks of Bethlehem devoted themselves more particularly to the care of the insane. Certainly Theodosius, the Cœnobiarch, built an infirmary for the sick in mind as well as in body near Bethlehem in the Holy Land about 529. It looks, also, as if patients out of their mind were received in Bishopsgate (the original site of the London dependency) before 1400, the year in which occurs the first documentary evidence of insane patients there, to the number of six. The Priory had, however, obtained the title of "hospital" as early as 1329, when Edward III. grants the Master and Brethren a licence to collect alms. In any case a number of patients were handed over to the monks of Bethlehem Priory by some king who "disliked to have lunatics near him." The site of this early asylum in which they were confined was in Trafalgar Square (to use modern language), near the statue of Havelock; and I place the date of the transfer between 1375-78, when Edward III. built mews for his falcons near St. Martin's Church. Bethlehem owned—this is a corroboration of my argument—houses on this site from before 1400 until 1830.

The Bethlehem Hospital of the pre-Reformation period was always struggling with poverty; it was plundered again and again by dishonest servants; its Masters were absentees and pluralists; the succession of monks was interrupted for years. But if the Certificate of the Confraternity is worth the value stamped on its face, the ideal of religious devotion never quite disappeared. There were often, we hope, physicians who remembered One of Whom it was said, "He is beside Himself," and devoted themselves in His Name to the mentally afflicted "with unceasing solicitude."

